# Autumn protogyny and spring protandry: Mechanisms and adaptive significance in a Japanese headwater frog, *Rana sakuraii*

**DOI:** 10.1371/journal.pone.0320076

**Published:** 2025-04-04

**Authors:** Tokio Miwa

**Affiliations:** Department of Environmental Science, Tokyo Gakugei University, Koganei, Tokyo, Japan; Guangxi University, CHINA

## Abstract

In temperate-zone vertebrates, almost all studies on protandry and protogyny are based on spring breeding migrations or breeding sites and are discussed in terms of the advantages for pairing. However, both autumn migrations and wintering conditions are also important for studies on these behaviors because, in some vertebrates, pairings occur at wintering sites before the emergence at the main breeding sites. Nevertheless, the significance of both autumn migrations and wintering conditions for pairing is scarcely recognized or studied. This study is the first to report on the mechanism and adaptive significance of “autumn protogyny and spring protandry” in a temperate-zone amphibian species. I investigated the annual migrations of *Rana sakuraii*, a Japanese early spring breeding (ESB) frog species, for over 21 years. This species migrates toward its breeding sites during autumn, hibernates either at the same breeding sites or close by, and breeds immediately upon emergence from hibernation. Protogynous autumn migrations and protandrous ESB migrations were clearly observed every year, depending on the same factor: the difference in threshold temperature for hibernation (which was higher for females than males). Frogs hibernated in groups under boulders in streams. Pairings began during autumn migrations and mostly ended during the aquatic hibernation period. Based on the results of this study and other related reports, I propose the hypothesis that temperate-zone ESB amphibians exhibit “autumn protogyny and spring protandry,” which are not independent and always occur together successively. Moreover, to explain the adaptive significance of these contrasting behaviors, I propose “the surefire pairing hypothesis”: these pairings occur more securely in two stages, before and after emergence at main breeding sites (i.e., “Early pairings” and “Normal pairings”). Such pairing advantages are provided by autumn protogyny and group hibernation for “Early pairings,” and by spring protandry for “Normal pairings.”.

## Introduction

Protandry (the earlier emergence or arrival of males at breeding sites) is widespread in the animal kingdom, having been reported from diverse taxa, including fish [1,2], amphibians [3,4], birds [5,6], a squirrel [7], and butterflies [8,9]. In contrast, protogyny (earlier emergence or arrival of females) is less common, with few reports of this phenomenon [10–12]. In temperate-zone vertebrates, both behaviors have been mostly studied based on spring breeding migrations or breeding sites and discussed in terms of the advantages for pairing (mating). However, there are only a few reports on the underlying mechanisms and adaptive significance of both behaviors [13,14]. Furthermore, in some vertebrates, most pairings occur at wintering sites before the emergence at the main breeding sites; for example, some anurans [15–23], many urodeles [24–26], and most migratory waterfowl [27–29]. Therefore, autumn migrations and wintering conditions are inevitably more important for studying both behaviors in the pairing of such vertebrates. However, the significance of both autumn migrations and wintering conditions for pairing is scarcely recognized. The present study is the first report on the mechanism and adaptive significance of amphibian protogyny, particularly of “autumn protogyny and spring protandry.”

**Fig 1:**
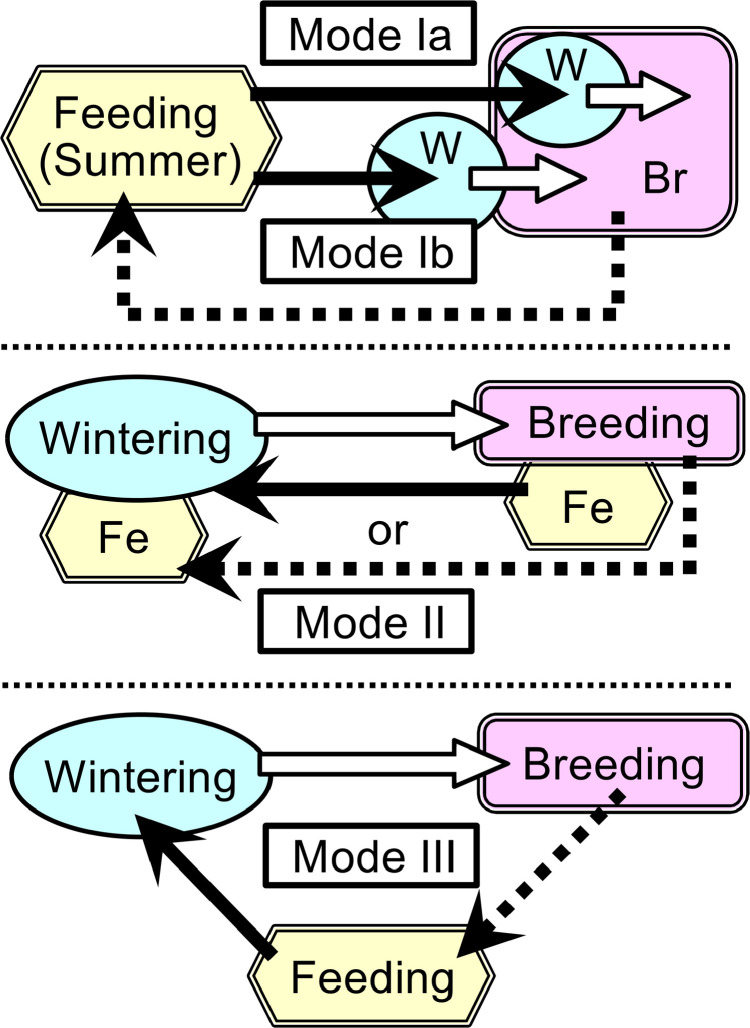
Annual migration modes of temperate-zone amphibians based on breeding, feeding, and wintering (hibernation) sites. Solid arrows: autumn migration. Open arrows: (spring or summer) breeding migration. Dotted arrows: summer post-breeding migration. W: wintering. Br: breeding. Fe: feeding. Mode I (top): most explosive-breeding amphibians (breeding over a short period in winter or early spring, with near-synchronous emergence at breeding sites immediately following emergence from hibernation), which hibernate at the aquatic breeding sites or close to the terrestrial sites, and their autumn migrations are pre-breeding migrations or purely breeding migrations for some species. Ia: aquatic hibernators (e.g., most urodeles, brown *Rana* spp., and some *Bufo* spp.); Ib: terrestrial hibernators (e.g., *Bufo* spp.). Modes II (middle), III (bottom): most prolonged breeding anurans (breeding over a long period from late spring to summer, or twice or more in a year; e.g., hylid frogs and green *Rana* spp.), some explosive-breeding anurans, and some urodeles, which hibernate at separate sites from their breeding sites. Their autumn migrations are post-breeding movements, allowing them to leave their breeding sites and prepare for wintering. Most migratory vertebrates usually migrate according to Mode II or III and perform pairings at breeding sites. However, most migratory waterfowl engage in pairings and copulation at wintering sites (Mode II) before spring breeding migrations [27–29]. Therefore, their autumn migrations are breeding migrations for pairings.

In temperate and subarctic zones, amphibians, particularly anurans, can be divided into two groups depending on their breeding patterns: “explosive breeders,” which breed over a short period in winter or early spring, with near-synchronous emergence at breeding sites immediately following emergence from hibernation, and “prolonged breeders,” which breed over a long period from late spring to summer, or twice or more in a year [30,31]. Their annual migration can be divided into three modes based on the habitats in which they breed, feed (summer), and hibernate (winter) (Fig 1). Mode I species (Fig 1) are generally explosive breeders (e.g., most urodeles, brown *Rana* spp., some *Bufo* spp.) and hibernate at or close to their aquatic breeding sites. Their autumn migrations are movements toward breeding sites and serve as either pre-breeding or purely breeding migrations for some species. Moreover, in many Mode I species, some or most pairings occur before emergence at the main breeding sites in spring. Therefore, their autumn migrations and wintering conditions are important for pairing. In contrast, Mode II and Mode III species (i.e., most prolonged breeding anurans and most other migratory animals, Fig 1) hibernate at sites other than their breeding sites. Their autumn migrations involve leaving breeding sites for wintering sites, but are not as essential for subsequent breeding.

In this paper, I focus on both the autumn and spring migrations of Mode I species. The common abbreviations and key terms for Mode I species, as used throughout this paper, are defined in Table 1. Many early spring breeding (ESB) amphibian species (i.e., Mode I species) exhibit ESB migrations (or the emergence days from hibernation) triggered by a rise in ambient temperature, unique to each species’ threshold values [32–36]. These migrations were considered to be protandrous (i.e., early emergence of males) [3,4,19,37–44]. Thus, it is inferred that their spring protandry occurs due to the difference of threshold values for emergence from hibernation (which were higher for females than males). However, detailed studies on such spring protandry (i.e., focusing on the specific differences in emergence days or triggering temperatures between the sexes) are very few [32,39,43,44]. Furthermore, although autumn migrations toward breeding sites are important for pairings, studies on these migrations are rare, particularly in anurans [39,45–51] and urodeles [37,52–55]. Notably, there is just one such report on whether these migrations represent protandry or protogyny [37].

**Table 1:** Definitions and explanations of abbreviations and key terms used in this paper.

Abbreviations and key terms	Definitions	Explanations
ESB	Early spring breeding	Breeding that occurs immediately after emergence from hibernation by Mode I species (Fig 1).
ESB amphibians	Early spring breeding amphibians	Amphibians that exhibit early spring breeding (i.e., Mode I species (Fig 1)).
ESB migrations	Early spring breeding migrations	Breeding migrations of Mode I species (Fig 1).
F, M, P	(Single) Females, (Single) Males, Pairs	–
AD	Arrival day	Day of arrival at or close to aquatic breeding sites during autumn migrations by ESB amphibians.
AD (F), AD (M)	Arrival day of (single) females, Arrival day of (single) males	–
ED	Emergence day	Day of emergence at the main aquatic breeding sites, immediately after emergence of hibernation by ESB amphibians.
ED (F), ED (M)	Emergence day of (single or amplectant) females, Emergence day of (single) males	–
Early pairings	–	Pairings performed before the ED. These occur in three stages: 1) during autumn or just before wintering; 2) during wintering (hibernation); and 3) immediately after emergence from hibernation but before emergence at the main aquatic breeding sites (more details in the Discussion section and Fig 6).
Normal pairings	–	Pairings that occur after the ED of females (more details in the Discussion section and Fig 6).
PMAT	Prerequisite minimum air temperature	The threshold temperature required to induce readiness for autumn migrations.
DCT	Daily cumulative temperature	In *R. sakuraii*, prerequisite DCT for the start of ESB migration was 22.4°C or more. For the three standards used to calculate the DCT, refer to Table 10 and Miwa [32].
GH, SH	Group hibernation, Solitary hibernation	–

Since December 1991, I have studied the annual migrations (classified as Mode Ia, Fig 1) of a Japanese headwater frog, *Rana sakuraii*, in detail. Previously, I investigated the factors influencing both autumn and ESB migrations of *R. sakuraii* and proposed common conditions responsible for the timing of these migrations in temperate-zone ESB amphibians (i.e., Hypotheses I and Ⅱ, Table 2) [36,51]. Building upon these findings and previous reports on other species [37,39,56], I further proposed Hypothesis Ⅲ (Table 2) [51]. Subsequently, based on Hypothesis Ⅲ and previous reports on amphibian spring protandry [3,4,19,37–44], I hypothesized that if ESB migration is protandrous (i.e., males have lower threshold values for hibernation compared to females), the autumn migration should be protogynous, as males would enter hibernation later than females, according to this hypothesis. Moreover, in many ESB anuran species (including *R. sakuraii*), it has been reported that some or most individuals engage in “Early pairings” [15–24,57]. Therefore, I presumed that “Early pairings” are highly dependent on the autumn migration and the associated wintering conditions. Therefore, in this study, I investigated sexual differences at the start, end, and emergence days of both migrations, focusing on the triggering factor (i.e., threshold temperature values for hibernation in each sex) and the timing of pairing. Here, I report on the autumn protogyny and spring protandry of *R. sakuraii*, identify the proximate factors responsible for these contrasting behaviors, and propose a hypothesis for the common underlying mechanism of these behaviors in ESB amphibians. Furthermore, I propose a hypothesis to elucidate the adaptive significance of these behaviors in ESB amphibians.

**Table 2:** Three hypotheses proposed as common conditions responsible for the timing of both autumn and early spring breeding (ESB) migrations in temperate-zone ESB amphibians, based on the previous results from *Rana sakuraii* and other related studies.

Hypothesis I [36]	The start of ESB migrations depends on three conditions during hibernation: (1) reaching the critical daily cumulative temperature (DCT), (2) the passing of the critical number of days (in effect, the increase in day length to a threshold), and (3) the rise in daily temperature to a threshold value after conditions (1) and (2).
Hypothesis Ⅱ [51]	The start of autumn migrations depends on three conditions: (1) the experience of a prerequisite minimum air temperature (PMAT), (2) the decrease in day length to a threshold value, and (3) the first rainy day (or day with high humidity) after conditions (1) and (2), regardless of the air temperature on the start days. The end of autumn migration depends on the ambient temperature dropping below a threshold value.
Hypothesis Ⅲ [51]	Based on the Hypotheses I and Ⅱ, the threshold value of ambient temperature inducing the start of the ESB migration is almost the same as that inducing the end of the autumn migration (i.e., the threshold for entering hibernation is the same as that for emerging from hibernation). Moreover, these values are approximately the same as the PMAT that induces the start of the autumn migration. In *R. sakuraii*, these three threshold values are approximately 5°C.

**Fig 2:**
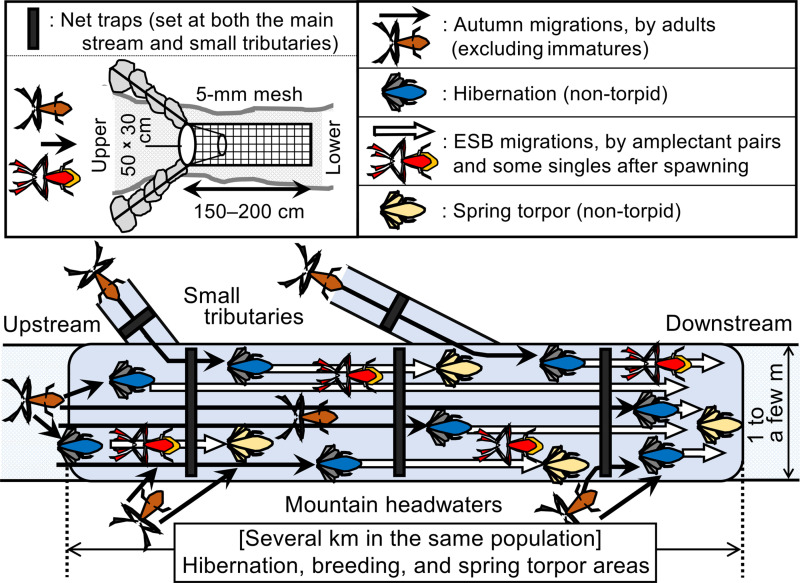
Schematic diagram of annual migrations in *Rana sakuraii* and a net trap (upper left). Solid arrows: autumn migrations from terrestrial sites into headwaters for hibernation and pairing (only adults, excluding immatures). Open arrows: ESB migrations (entirely underwater), for spawning and spring torpor (primarily by pairs and some singles after spawning). The autumn and ESB migrations distances vary widely between individual frogs, ranging several tens of meters to several kilometers [57,58]. The summer migrations, showing the return to terrestrial upper sites, are not depicted.

**Fig 3:**
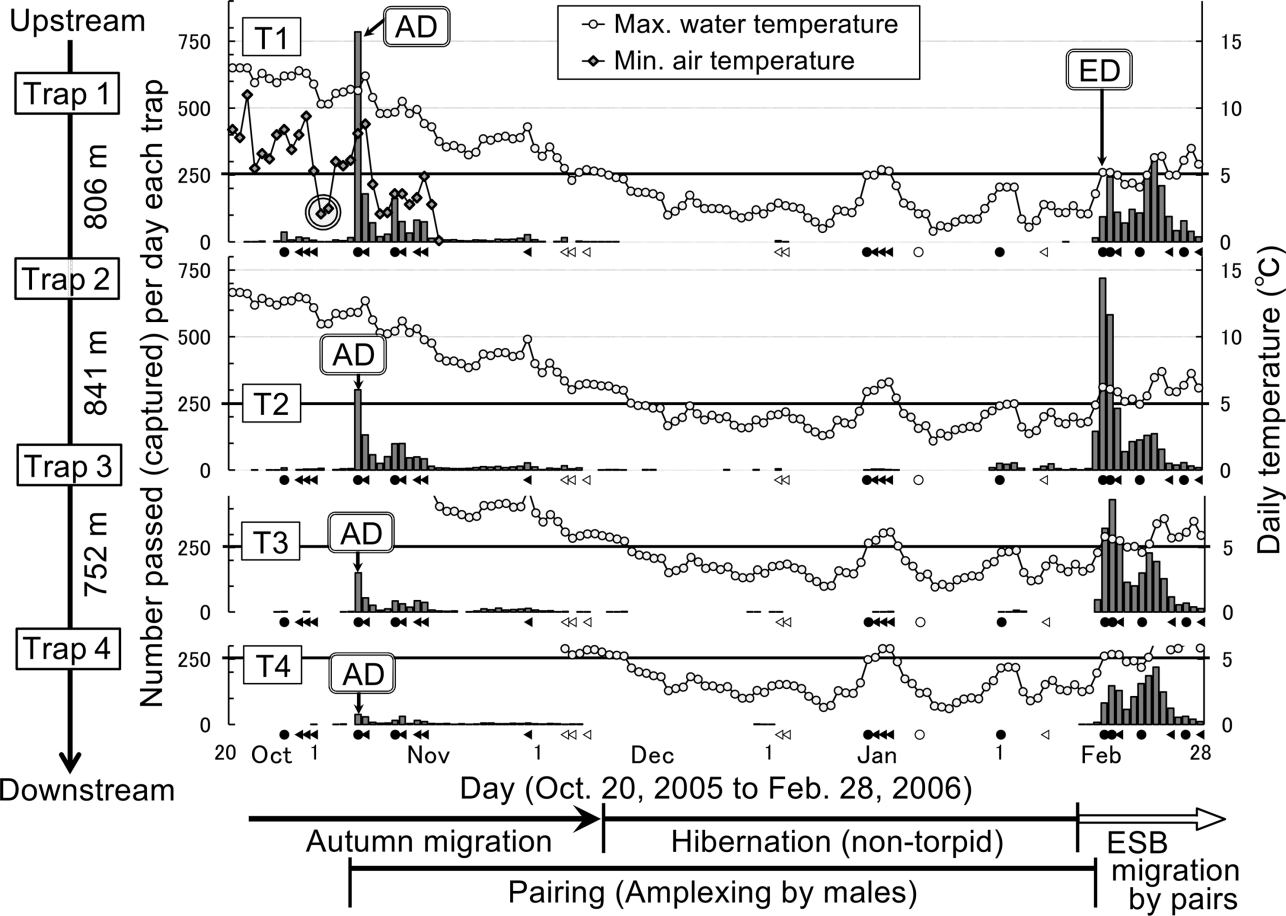
Typical annual migratory patterns of *Rana sakuraii*, including movements during the hibernation period, with comparisons of migration movements among upper, middle, and lower stream positions. These data were collected across upper, middle, and lower stream positions, from in-stream traps set at consistent locations during the representative seasonal year 2005–2006. The distances between traps are as follows: Trap 1 (T1) to Trap 2 (T2), 806 m; Trap 2 (T2) to Trap 3 (T3), 841 m; Trap 3 (T3) to Trap 4 (T4), 752 m. AD: arrival days. ED: emergence days. Double circle: prerequisite minimum air temperature (PMAT) that induces the start of autumn migrations. The bold line at 5°C along the daily maximum water temperature axis indicates the threshold between the end of autumn migrations and the beginning of spring migrations. Symbols: ●, rain; ▲, light rain; ○, snow; **∆ **, light snow.

**Fig 4:**
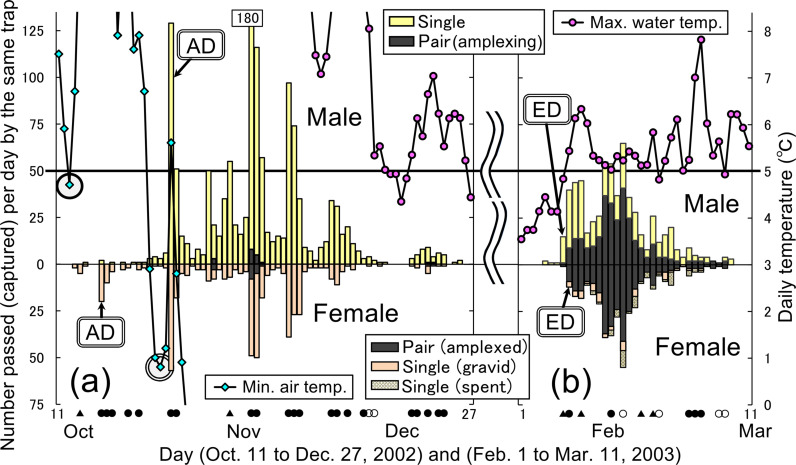
Typical migratory patterns of male and female *Rana sakuraii* during autumn (a) and ESB migrations **(b), based on data from an in-stream trap set at the midstream position during the representative seasonal year 2002–2003.** The figure shows males (upper) and females (lower) collected by the same trap set at the midstream site in 2002–2003. (a). In the autumn migration, females arrived at aquatic breeding sites earlier than males and ended instream-movements earlier than males, and some males began amplexing females (i.e., pairing). Single circle: prerequisite minimum air temperature (PMAT) for females; double circle: PMAT for males. (b). In the ESB migration, single males emerged earlier than single or amplexed females. When the ESB migrations began and frogs emerged, most females had already paired. Moreover, amplectant pairs maintained amplexus and migrated for a few days to several weeks because of difficult spawning. The upper part of panel (b) includes single males, both before and after ejaculation, as well as those that released the amplexus before female spawning due to fatigue from prolonged amplexus (unpublished data). Accordingly, most single gravid females observed during the ESB migration (lower part of b) were individuals that were released from male-amplexus before female spawning (unpublished data). Symbols as described in Fig 3.

**Fig 5:**
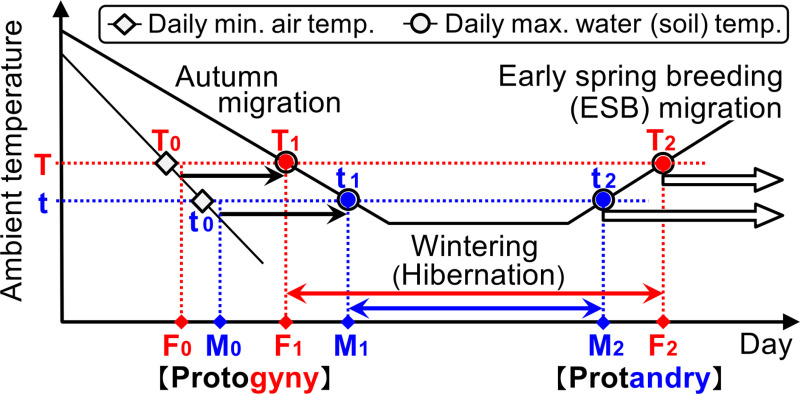
Mechanism of autumn protogyny and spring protandry in *Rana sakuraii* and other temperate-zone ESB amphibians. Solid black arrows: autumn migration. Open black arrows: ESB migration. Red and blue arrows: wintering periods for females and males, respectively. F_0_, M_0_: start days of the autumn migration (i.e., the entering and arrival days at aquatic breeding sites from terrestrial sites) for females and males, respectively. F_1_, M_1_: complete end days of the autumn in-stream movements (i.e., the days preceding hibernation) for females and males, respectively. M_2_, F_2_: emergence days from hibernation (i.e., the start of ESB migration) for males and females, respectively. T and t: threshold value**s** for hibernation for females and males (T >  t), respectively (T_0_ ≈  T_1_ ≈  T_2_; t_0_ ≈  t_1_ ≈  t_2_). T_0_ and t_0_: thresholds inducing readiness for autumn migration (i.e., required PMAT before the start of autumn migration); T_1_ and t_1_: thresholds inducing hibernation; T_2_ and t_2_: thresholds inducing emergence. Male and female frogs move into streams from terrestrial sites on the first rainy days after experiencing the PMAT (i.e., thresholds: T_0_ and t_0_ or lower) [51], and each frog completes in-stream movements and remains under boulders when maximum water temperatures fall to or below the thresholds T_1_ and t_1_ [51]. Subsequently, each frog emerges and migrates when the maximum water temperatures rise to or above the thresholds T_2_ and t_2_ [36]. In the case of terrestrial (not aquatic) hibernation, daily water temperature refers to soil (ground) temperature.

## Materials and methods

### Study area and species overview

This field study began in 1991 and was conducted in the mountain headwaters of the upper basin of the Akigawa River (35°40’N and 135°9’E), located approximately 50 km west of Tokyo, Japan. The headwaters originate from the mountain at 1,019 m and are characterized by clear, narrow, and rapidly flowing waters with rocky banks [50,57,58]. The main research area (approximately 2,500 m long) was selected along the stretches of the headwaters, at altitudes ranging from 300–500 m [36,50,57].

*Rana sakuraii* is one of the most abundant and migratory anuran species in Japan. Its habitat is restricted to mountain headwaters, which are cool, clear, and rapidly flowing streams ranging from elevations as low as 150 m to headwaters reaching up to 1,500 m. These frogs are widely distributed throughout Honshu Island (the main island of Japan), excluding the northern region [57,58]. The frogs live at terrestrial sites around mountain headwaters in summer. In autumn, they migrate from terrestrial habitats into streams and swim downstream underwater to hibernate (non-torpid state) under boulders in running water. In early spring, they emerge from hibernation, migrate downstream, and spawn (lay eggs) behind boulders or in gaps among rocks in stream pools. Thereafter, they remain still under boulders in shallow sites during the spring torpor period (non-torpid state). In summer, they migrate back upstream to the headwaters (Fig 2). Thus, the annual life cycle of *R. sakuraii* can be divided into five periods: 1) autumn migration (for hibernation and pairing by all adults, excluding immatures), 2) hibernation, 3) ESB migration (for spawning and spring torpor, primarily by pairs and some singles after spawning), 4) a spring torpor period, and 5) a summer active period (for returning to terrestrial sites around the source of headwaters) [57,58] (Fig 2). Throughout the aquatic phase of their life periods (i.e., from the autumn migration into streams to the spring torpor period), *R. sakuraii* consistently dwell underwater, swimming exclusively at the bottom of the streams. Therefore, during this aquatic phase, the skin on their head, body, legs, and arms becomes remarkably loose, forming pleats, and the webs become thicker and broader to increase skin respiration and adapt to hypoxic conditions [57,58].

A particularly notable yet potentially misunderstood characteristic of this species is that, within the same population, the aquatic hibernation, breeding, and spring torpor sites are nearly the same and span several kilometers [57,58] (Fig 2). Notably, spawning sites for the same population are not confined to specific stream pools but are distributed across several tens or even hundreds of pools within the same stream (spaced approximately every several tens of meters from upper to lower sites) [57,58] (Fig 2). Consequently, the ESB migration distances vary widely among pairs, ranging from several tens of meters to several kilometers [57,58] (Fig 2). This variability stems from the significant challenges females face during spawning; if amplectant females cannot spawn promptly, they will continue migrating downstream for several days to a few weeks [57,58]. Thus, most pairings (i.e., amplexus) in *R. sakuraii* typically persist for extended periods [57,58].

### Study period and data collection

Frog behaviors were investigated over a span of 21 years, as follows: autumn migration was studied across six years (1999, 2001, 2002, 2005, 2013, and 2014) from mid-October to late December. Wintering behavior and ESB migration (spawning migration) were observed over 21 years (1991–1992, 1992–1993, and 1998–1999 to 2016–2017), from early December to early February for wintering, and from late January to mid-March for ESB migration (refer to S1 Table).

To study both autumn and ESB migrations, frogs migrating underwater were captured using net traps (constructed using a 5 mm mesh, with an elliptical entrance approximately 50 cm at the major axis and 150–200 cm in length) [36,50,51,57] (Fig 2). These traps were strategically set at narrow points along the stream where the currents were rapid. Specifically, the traps were set upstream, downstream, and in small tributaries along the same stream [36,51,57] (Fig 2). The number of traps deployed varied each year depending on the environmental conditions (mainly stream water levels and snowfall). Traps were checked and emptied at least once daily at around 0800 h. Captured frogs were placed into buckets, identified by sex, counted for amplectant pairs and singles (males and gravid or spent females), and released downstream of the traps [36,51,57]. Moreover, over three years (2001–2002, 2002–2003, and 2005–2006), to study movements during the mid-hibernation period, net traps were set from autumn through the ESB migration periods and examined every day. Furthermore, to study wintering behavior, frogs hibernating under boulders in shallow streams were captured using hands or dip nets.

In addition, efforts were made to minimize harm to the trapped frogs in the net traps as frogs are vulnerable to high water pressure and low temperature over a long exposure period. Therefore, the measurement process included: 1) daily retrieval of frogs from traps, and if possible, multiple times per day; 2) setting adjacent traps at intervals of at least 750 m to minimize the likelihood of frogs re-entering traps; and 3) constructing long traps (150–200 cm) to reduce the water pressure within them (Fig 2).

Both water and air temperatures were recorded every 10 min to the nearest 0.1°C using Thermo Recorder TR-52 data loggers (T and D Corporation, Matsumoto, Japan). Water temperature was measured near the upstream side of almost every trap as the temperature varied by 0.5–2.5°C between the main stream and the small tributaries as well as between the upstream and downstream points within the study area. The sensors were placed at depths of approximately 20–30 cm in gaps beneath submerged boulders in rapidly flowing areas. Air temperature was measured at two upstream sites within the study area, from approximately 10 cm above the ground [36,50,51,57].

### Definitions of the three days: arrival, moving end, and emergence

In this study, “arrival days” refer to the first major days when frogs were captured during autumn migrations, and “emergence days” denote the first major days when frogs were captured during ESB migrations [36,51]. This distinction is necessary because: 1) all traps were set within the broad breeding sites, not at their boundaries (Fig 2), and 2) even after the arrival or emergence at breeding sites, most frogs continue migrating, with distances differing widely among individuals during autumn migrations and among pairs during ESB migrations (Fig 2). Therefore, many captured individuals in each trap likely migrated from upper streams after the arrival or emergence days. This point is often misunderstood because, for most migratory animals, trapping data set at the boundaries of the breeding sites during breeding migrations typically represent the arrival or emergence days of all individuals at the breeding sites by the day of the last capture in the traps.

Subsequently, based on these field observations, the three key days (arrival, moving end, and emergence) were defined as follows. The “number captured per day” for both autumn and ESB migrations was defined as the number of frogs captured in the 24-h period between 0800 h and 0800 h, as frogs mostly stopped moving at 0800 h during both migrations [36,50]. For the autumn migration study, the “arrival day (i.e., start day)” refers to the day when frogs entered the aquatic breeding sites from terrestrial habitats and were captured by in-stream traps. This “arrival day” was defined as the first day when the number of frogs captured per day in each trap was 1% or more than the total number captured in that year in each trap [51]. Similarly, the “in-stream moving end day” refers to the day before starting hibernation, defined as the last day during which at least one frog was captured in each trap, with no captures for the subsequent five consecutive days [51]. For the ESB migration study, the “start day” refers to the day of emergence, defined as the first day during which the number of frogs captured per day in each trap was 1% or more than the total number captured in that year in each trap [36].

### Re-entering individuals and migration distances

During both the arrival and emergence days, individuals (that were trapped in the upper traps and released downstream) never re-entered other traps. This is because: 1) frogs cannot move upstream due to the rapid flows and cannot move on land during these periods due to the freezing air temperatures, 2) frogs cannot migrate over long distances in a single day. Although some frogs migrated long distances ( > 1–2 km) over the entire duration of ESB migrations, their average migratory distance was several hundred meters, with daily distances typically being only a few dozen meters to a few hundred meters at most (unpublished data). Therefore, the traps were set at intervals of at least 750 m to minimize re-entry, and 3) the arrival or emergence days coincide closely across several traps, with differences of only a few days, making it unlikely that the frogs re-enter the traps downstream on the same or next day.

Primarily, during both the migration periods, the captured frogs from the same trap (except for those in the lowest traps) were given the same mark by punching holes (1.5 mm in diameter) at two or three sites through the broad webs of the hind limbs, allowing for individual discrimination. These web punches were regenerated by the next year. Importantly, this marking procedure did not interfere with amplectant pairings or their migrations.’

### Statistical analysis

In previous studies, it was found that both migrations were significantly affected by temperature conditions as follows: during the autumn migration, arrival (start) days consistently coincided with the first rainy days after experiencing PMAT, regardless of the air temperature on those days. PMAT should be approximately 5°C or lower [51]. Moreover, the days of ending in-stream movements were significantly affected by the daily maximum water temperature (the threshold of 5°C) [51]. In the ESB migration, the start (emergence) days were significantly affected by the rising daily maximum water temperature to the threshold of 5°C after the reaching the critical daily cumulative temperature (DCT) (e.g., a total of 22.4°C from January 20 each year, cumulative of the effective daily temperatures as values over 4°C of the daily maximum (not merely over 0°C) [36].

Based on the aforementioned findings, first, the study compared sexual differences of three days and three temperatures on 1) the arrival, 2) the in-stream moving end during the autumn migrations, and 3) the emergence (start) during the ESB migrations, using the Wilcoxon signed rank tests. However, the temperature of the emergence (start) days was analyzed by excluding the data from the year 2007 due to exceptionally warm weather conditions (refer to the results). Second, the threshold values of the three temperatures were calculated by using the resulting logistic regression equations for each sex, following methods established in previous studies (for more details, refer to Miwa [36,51]).

### Ethics statements

Field work and frog handling were approved by the Akigawa River Fishery Cooperative and Hinohara Village office. In particular, setting net-traps in the streams was conducted under the license of the Akigawa River Fishery Cooperative. Other field permits were not required for this specific study. All methods were carried out in accordance with the Animal Experimentation Guidelines of Tokyo Gakugei University. In addition, this study did not involve or affect any endangered species.

## Results

### General patterns of both migrations and outlines of pairing timing

The start days of autumn migration (i.e., arrival days of the frogs into the aquatic breeding sites from terrestrial sites) always coincided with the first rainy days after the minimum air temperature had dropped to the PMAT, that is approximately 5°C or lower (Figs 3 and 4a). Except for the arrival days, most frogs usually did not migrate downstream for long distances. However, some frogs swam downstream on rainy days (Figs 3 and 4a). Particularly, in 2002, even after the arrival, many frogs moved downstream due to the frequent heavy rainfall that autumn (compare Fig 4a with the 2005 autumn migrations shown in Fig 3). During autumn migrations, more frogs passed through upstream traps than downstream traps every year (Fig 3). Autumn in-stream movements ended completely in early or mid-December when the maximum water temperature dropped below 5°C (Figs 3 and 4a), prompting frogs to hibernate in groups under boulders in running water. During hibernation, the frogs were always reactive and non-torpid, so that some frogs moved and entered the traps (Fig 3). Moreover, the frogs swam quickly when I lifted boulders to attempt capturing them, despite the very low water temperatures (0–4°C). When the daily maximum water temperature rose to approximately 5°C or more, the frogs emerged from hibernation and started ESB migrations downstream (Figs 3 and 4b). These ESB migrations were mainly for spawning by amplectant pairs (Fig 4b). Amplectant pairs spawned behind boulders and then separated. Subsequently, single frogs of both sexes migrated for short distances and remained still in shallow sites during the spring torpor period.

Every year, in the early period of autumn migrations, some males began amplexing females (i.e., pairing) (refer to the four days indicated by black rectangles in middle section of Fig 4a). The mean start day of male amplexing was 36.2 (day 1 =  October 1) (Table 3). These start days coincided with the arrival days (i.e., the days they entered the breeding streams) or occurred several days after the arrival (Table 3). This timing corresponded with daily water temperatures dropping to under approximately 10°C (Table 3) and the males’ body color changing to nuptial coloration. Percentage of amplexed females (i.e., paired females) to the total females was 6.5% or more during the autumn migration period from the start of pairing days to the end of the migratory movements (Table 4). Subsequently, the rates of amplexed females increased, and by the end of the non-torpid hibernation period (i.e., just before the start of spring migration), approximately half of the females were amplexed by males (Table 4). Moreover, when the ESB migrations began and frogs emerged, most females had already paired (88.7%, Table 4). Furthermore, during the 21-year study period of ESB migrations, I observed several tens of thousands of frogs in total through visual observations. However, only four single gravid females were observed, while the rest were either single males or amplectant pairs. Almost all the females were paired before emergence and arrived at the main breeding sites (stream pools). Thus, during the ESB migration period, observing single gravid females was extremely rare.

**Table 3:** Start days and temperatures of male *Rana sakuraii* pairing (amplexing behavior) during autumn migratory movements, after arrival at the breeding streams.

Year	Male arrival			Start of male amplexing		
	Day	Water temp. (°C)		Day	Water temp. (°C)	
	(1 = Oct. 1)	max.	min.	(1 = Oct. 1)	max.	min.
1999	28.0	11.6	13.5	42	10.5	9.5
2001	22.0	12.5	13.5	36	10.6	10.1
2002	32.3	10.6	11.5	36	9.5	8.0
2005	34.6	12.0	12.7	37	9.3	8.8
2013	35.0	10.8	9.9	35	10.8	9.9
2014	31.0	10.9	9.3	31	10.9	9.3
Mean	30.5	11.4	11.7	36.2	10.3	9.3

**Table 4:** Percentage of amplexed *Rana sakuraii* females (paired females) relative to total females during the three key aquatic life periods from autumn instream movements to early spring breeding (ESB) migrations in the breeding streams.

Autumn migration^a^		Hibernation^b^								ESB migration
Late Oct. to mid-Dec.		December		January			Early to mid-February			February
Total (from the first arrival day to the end)	From the first amplexed day to the end	11–20	21–31	1–10	11–20	21–31	1 Feb. to 7 days before the end	4–6 days before the end	1–3 days before the end	Start to peak (i.e., emergence time)
4.5%	6.5%	6.7%	11.6%	14.6%	18.3%	19.9%	24.6%	36.3%	55.6%	88.7%

Regarding the data for both autumn and ESB migration periods, all information is based on net-trapped individuals. The data for the hibernation period is based on individuals captured by hands or dip nets. For more detailed yearly data, refer to S2–4 Tables.

a,b It is important to note that the values for both autumn migration and hibernation periods are likely higher than reported. This discrepancy in (1) autumn migration data can be attributed to amplexed females remaining stationary without migrating. Therefore, most pairs during late autumn migration periods likely did not enter the instream net-traps. (2) the hibernation data is due to it representing only a small portion of the population because it was derived only from sites where capture by hand was possible. Approximately 85% of adults, as estimated from recapture data, hibernate in locations that are inaccessible to researchers (e.g., under large rocks that could not be moved, or beneath large boulders in deep stream pools). These individuals were expected to exist in groups or as amplectant pairs, whereas individuals that are feasibly captured generally hibernate solitarily. Moreover, in late January, if single males and females captured by hands or dip nets were kept together in a bucket, most females were usually amplexed by males shortly after.

In addition, during the late ESB migration periods each year, some amplectant males released the females before successful spawning due to fatigue caused by both long-term amplexus and stream water pressure (unpublished detailed data). Particularly, inside the stream traps, some amplectant males released the females before female spawning (unpublished detailed data). However, single males were unable to amplex females due to the water pressure in rapid streams.

I captured a total of 14,003 autumn migrating adults over six years, and 89,817 ESB migrating adults and 29,397 hibernating adults over 21 years (refer to S1 Table).

### Differential responses of males and females in relation to the arrival and end days of autumn migrations

Over the six-year study period, females consistently arrived at aquatic breeding sites significantly earlier than males each year (mean difference: 6.9 days (n =  6 years, z =  2.201, P =  0.031; n =  22 traps, z =  4.069, P <  0.001)) (Table 5 and Fig 4a). The mean arrival days were 23.6 (day 1 =  October 1) for females and 30.5 for males (Table 5). Moreover, PMATs for females were significantly higher than those for males (mean difference: 1.60°C (n =  6, z =  -2.108, P =  0.063; n =  22, z =  -3.910, P <  0.001)) (Table 5). The mean PMAT of the arrival was 4.75°C for females and 3.15°C for males (Table 5). Logistic regression equations yielded threshold PMAT values of 5.76°C (females) and 4.23°C (males) for the arrival (Tables 5 and 6).

**Table 5:** Arrival days at breeding sites and prerequisite minimum air temperature (PMAT, °C) that induces readiness for the autumn migration in male and female *Rana sakuraii.*

	Year	Traps	Arrival day (1 = October 1)			PMAT (°C)		
		*n*	Male	Female	D [F - M]^a^	Male	Female	D [F - M]^a^
	1999	1	28.0	27.0	-1.0	3.10	3.10	0.00
	2001	3	22.0	17.0	-5.0	4.70	5.10	0.40
	2002	8	32.3	18.5	-13.8	0.80	4.70	3.90
	2005	5	34.6	29.8	-4.8	2.78	4.78	2.00
	2013	2	35.0	29.0	-6.0	4.60	4.90	0.30
	2014	3	31.0	20.0	-11.0	2.90	5.90	3.00
Total	6	22						
Mean			30.5	23.6	-6.9	3.15	4.75	1.60
P (6 yrs.)^b^					0.031			0.063
P (22 traps)^b^					< 0.001			< 0.001
Threshold^c^						4.23	5.76	1.53

^a^Differential values that subtract males from females.

^b^P values of Wilcoxon signed rank tests on the sexual difference.

^c^Threshold values calculated using logistic regression equations (refer to Table 6).

**Table 6:** Results of logistic regression analyses for factors controlling the start of autumn migrations and the threshold values of prerequisite minimum air temperature (PMAT, °C) inducing readiness for autumn migration in *Rana sakuraii.*

Sex	n^a^	Coefficients		Constant	R^2^	Predictive accuracy	AIC	Threshold of PMAT (°C)^b^
		*X*_1_	*X*_2_				(i.e., *X*_2_ (°C) when *Y* = 0.5)	
Male	635	12.082^***^	-1.850^***^	-4.256^***^	0.715	96.5%	150.87	4.23
Female	635	5.658^***^	-0.579^***^	-2.327^***^	0.545	92.1%	312.11	5.76

The response variable (*Y*): daily migration on a particular day (yes =  1; no =  0). The explanatory variables: *X*_1_ (daily weather: rain =  1; no rain =  0) and *X*_2_ (minimum values of daily minimum air temperatures (°C) recorded through the day of analysis).

^a^n (635): the total number of days from the first day when the daily minimum temperatures fell to under 10.0°C, to the first peak day for all 22 traps.

^b^Threshold values (i.e., *X*_2_ when *Y* =  0.5) were calculated as *X*_1_ =  1 (the rainy days). ^***^P <  0.001 (Wald chi-square test). For more details, refer to Miwa [51].

Thus, protogyny occurred based on the difference in the threshold temperature for hibernation, which was higher for females than males. Consequently, the degree of protogyny (i.e., difference in the number of days between male and female arrival timing) fluctuated depending on the falling tendency (gradual or sudden) of daily air temperatures each year.

Similarly, females completed autumn in-stream movements (i.e., entered their hibernation period) significantly earlier than males each year (mean difference 7.5 days; n =  6, z =  2.201, P =  0.031; n =  22, z =  4.069, P <  0.001) (Table 7). The mean end days were 72.1 (day 1 =  October 1) for females and 79.6 for males (Table 7). Moreover, the end temperatures (daily maximum water) for females were significantly higher than those for males (mean difference 0.64°C; n =  6, z =  -2.201, P =  0.031; n =  22, z =  -3.962, P <  0.001) (Table 7). The mean temperatures on the end day were 6.10°C for females and 5.45°C for males, and those on the following day were 5.30°C and 4.60°C, respectively (Table 7). From the results of logistic regression equations, the threshold values were calculated as 5.82°C for females and 5.16°C for males (Tables 7 and 8).

**Table 7:** End days and daily maximum water temperatures that trigger the end of autumn in-stream movements in male and female *Rana sakuraii.*

	Year	Traps	End day (1 = October 1)			End temp. (°C)		
		*n*	Male	Female	D [F - M]^a^	Male	Female	D [F - M]^a^
	1999	1	81.0	80.0	-1.0	4.80	5.70	0.90
	2001	3	77.7	65.3	-12.3	5.40	6.23	0.83
	2002	8	84.3	77.5	-6.8	5.53	6.07	0.54
	2005	5	75.6	72.6	-3.0	5.24	5.86	0.62
	2013	2	81.0	71.0	-10.0	5.85	6.35	0.50
	2014	3	78.3	66.3	-12.0	5.90	6.37	0.47
Total	6	22						
Mean			79.6	72.1	-7.5	5.45	6.10	0.64
P (6 yrs.)^b^					0.031			0.031
P (22 traps)^b^					< 0.001			< 0.001
Next day						4.60	5.30	0.70
Threshold^c^						5.16	5.82	0.66

^a^Differential values that subtract males from females.

^b^P values of Wilcoxon signed rank tests used to assess the sexual difference.

^c^Threshold values calculated using logistic regression equations (refer to Table 8).

**Table 8:** Results of logistic regression analyses for daily maximum water temperature controlling the end of autumn in-stream movements and the threshold values in *Rana sakuraii.*

Sex	n^a^	Coefficient	Constant	R^2^	Predictive accuracy	AIC	Threshold (°C) for the end
		*X*					(i.e., *X* (°C) when *Y* = 0.5)
Male	440	2.715^***^	-14.019^***^	0.554	86.6%	256.45	5.16
Female	440	2.217^***^	-12.897^***^	0.566	89.8%	234.44	5.82

The response variable (*Y*): daily migration (≥1 frog per day: yes =  1; no =  0). The explanatory variable (*X*): daily maximum water temperature (°C).

^a^n (440) indicates the total number of 20 days (10 days each, before and after the end day) in all 22 traps. ^***^P <  0.001 (Wald chi-square test).

### Differential responses of males and females in relation to the start day of ESB migrations

In the first year (1992), the start day and temperature could not be determined because the net traps were set too late to measure data for that year. Single males emerged and started migrations significantly earlier than the amplexed or single gravid females did (mean difference 2.5 days; n =  20 years, z =  -3.818, P <  0.001) (Table 9 and Fig 4b). The mean start days were 9.7 (day 1 =  February 1) for males and 12.2 for females (Table 9). Start days for both sexes varied widely from late January to early March and varied significantly over the 20-year study period (F_19, 62_ =  1.757, P <  0.001; Table 9), depending on when the water temperature reached the threshold of approximately 5°C after reaching the critical DCT. However, in 2007, the start days were in late January and start temperatures were > 6°C for each sex and trap (Table 9), due to exceptionally warm water conditions caused by heavy rain similar to an out-of-season typhoon that occurred on December 26–27, 2006. Therefore, the water level was high, and the water temperature was 5–8°C throughout January 2007. Consequently, the 2007 ESB migration commenced solely due to the critical DCT, as temperatures did not drop below the 5°C threshold.

**Table 9:** Start days and daily maximum water temperatures for early spring breeding (ESB) migrations (i.e., emergence days from hibernation) in *Rana sakuraii.*

	Year	Start day (1 = February 1)				Start temp. (°C)			
		n^a^	Male	Female	D [F - M]^c^	n^b^	Male	Female	D [F - M]^c^
	1993	8	5.0	6.1	1.1	2	5.00	5.35	0.35
	1999	5	14.8	17.8	3.0	2	4.85	5.00	0.15
	2000	5	8.4	12.8	4.4	3	4.77	4.90	0.13
	2001	5	15.4	15.4	0.0	5	5.06	5.06	0.00
	2002	10	2.2	3.7	1.5	9	4.78	5.03	0.26
	2003	9	8.2	9.1	0.9	8	4.93	5.39	0.46
	2004	4	14.3	15.5	1.3	4	5.03	5.25	0.23
	2005	4	10.0	11.3	1.3	4	5.03	5.35	0.33
	2006	5	8.8	11.8	3.0	5	4.94	5.30	0.36
	2007	4	-3.3	0.0	3.3	4	6.25	6.65	0.40
	2008	2	26.5	30.0	3.5	2	4.80	5.00	0.20
	2009	2	2.0	2.0	0.0	2	5.10	5.10	0.00
	2010	2	9.0	10.0	1.0	2	4.70	4.90	0.20
	2011	2	17.5	19.0	1.5	2	5.00	5.40	0.40
	2012	2	14.0	14.0	0.0	2	5.05	5.05	0.00
	2013	2	3.0	8.0	5.0	2	5.05	5.20	0.15
	2014	3	2.7	17.0	14.3	3	5.33	5.50	0.17
	2015	3	14.7	16.7	2.0	3	4.87	5.23	0.37
	2016	3	10.7	12.7	2.0	3	5.03	5.20	0.17
	2017	2	11.0	11.5	0.5	2	4.95	5.20	0.25
Total	20	82				69			
Mean			9.7	12.2	2.5		4.96^d^	5.18^d^	0.22
SD			6.8	6.7	3.1		0.15^d^	0.18^d^	0.14
P (Wilcoxon)^e^					< 0.001				< 0.001
P (ANOVA)			< 0.001	< 0.001			0.196^d^	0.108^d^	
Previous day							4.41^d^	4.61^d^	0.20
Threshold^f^							4.88^d^	5.10^d^	0.22

^a^Number of traps, including traps without data loggers for collecting water temperatures.

^b^Number of traps with data loggers.

^c^Differential values that subtract males from females.

^d^Values calculated excluding the year 2007, which was an extraordinarily warm year.

^e^P values of Wilcoxon signed rank tests on the sexual difference.

^f^Threshold values calculated using logistic regression equations (refer to Table 10).

Start temperatures for females were significantly higher than those for males (mean difference 0.22°C; n =  20 years, z =  -3.819, P <  0.001; Table 9). During the 19 years of study (excluding 2007), the mean start temperatures were 4.96°C for males and 5.18°C for females (Table 9). However, these start temperatures of either sex were not significantly different across the 19 years (F_18, 46_ =  1.833, P =  0.196, males; 0.108, females; Table 9). Logistic regression equation analysis revealed that the threshold values of the start temperatures were 5.10°C for females and 4.88°C for males (Tables 9 and 10). Moreover, each threshold value was almost the same as the respective thresholds for the end of autumn migration (Tables 7 and 8). Furthermore, each value was almost the same as the respective thresholds of the required PMAT before the start of autumn migrations, that is, the thresholds inducing readiness for autumn migration (Tables 5 and 6).

**Table 10:** Results of logistic regression analyses for factors controlling the start of early spring breeding (ESB) migrations (i.e., emergence from hibernation) and threshold values of the daily maximum water temperature in *Rana sakuraii.*

Sex	n^a^	Coefficients		Constant	R^2^	Predictive accuracy	AIC	Threshold (°C) for the start
		*X*_1_	*X*_2_				(i.e., *X*_1_ (°C) when *Y* = 0.5)	
Male	1300	2.326^***^	0.655^***^	-26.015^***^	0.672	91.2%	572.05	4.88
Female	1300	2.043^***^	0.532^***^	-22.315^***^	0.643	91.2%	629.00	5.10

The response variable (*Y*): daily migration (yes =  1; no =  0). The explanatory variables: *X*_1_ (daily maximum water temperature (°C)) and *X*_2_: (DCT: Daily cumulative temperature (°C)).

^a^n (1300): the total number of 20 days (10 days each, before and after the start day) of all 65 traps (excluding the four traps in 2007). Threshold values (i.e., *X*_1_ when *Y* =  0.5) were calculated as *X*_2_ (the DCT value) =  22.4 for all 65 traps. ^***^P <  0.001 (Wald chi-square test). Three standards for the DCT calculation were as follows: 1) daily temperature was used as daily maximum (not minimum or mean); 2) effective values of daily temperatures have used a value over 4°C (not merely over 0°C); and 3) reference dates for DCT (i.e., start dates for the calculation) were not fixed as a particular day because these dates fluctuated depending on the weather conditions in each year. Instead, at this time, the DCT value on the start days was set as 22.4°C, and the DCT values for the relevant day were calculated. For example, if the maximum water temperature was 5.2°C on the start day of a trap, the DCT on the previous day was calculated as 21.2°C (=22.4 – (5.2 – 4.0)); moreover, if the maximum was 5.5°C on the next day, the DCT was calculated as 23.9°C (=22.4 +  (5.5-4.0)). Regarding these standards, refer to Miwa [36].

Thus, protandry also occurred based on the difference in the threshold temperature for emergence from hibernation. Therefore, the degree of protandry (i.e., the number of days in the sexual differences in emergence timing) also fluctuated depending on the rising tendency (i.e., gradual or sudden) of daily water temperatures each year. For example, in three years (2001, 2009, and 2012) when the daily water temperatures showed a sharp rise from that of the previous day and reached the threshold values, both sexes emerged on the same days (Table 9). In contrast, females exhibited a longer delay in emergence (e.g., in 2014, Table 9) when the water temperatures abruptly dropped and remained low for several days due to heavy snowfall immediately after the males started to migrate.

## Discussion

### Mechanism of autumn protogyny and spring protandry in ESB amphibians

In my previous studies, I utilized mixed data without differentiating the sex of *R. sakuraii* to identify the driving factors of three key days: day 0, the start of autumn migrations (i.e., the transition from terrestrial to aquatic hibernation and breeding sites); day 1, the end of autumn migrations (i.e., entering hibernation); and day 2, the start of ESB migrations (i.e., emerging from hibernation) (Fig 5). Notably, day 0 occurred on the first rainy days after experiencing the PMAT (approximately 5°C or lower), regardless of the air temperature on the start days; day 1 was induced by the fall in daily maximum water temperature to a threshold value of 5°C or lower, and day 2 was induced by the rise in daily maximum water temperature to a threshold value of 5°C or higher [36,51]. These three threshold values were consistently near 5°C. Based on these findings and previous reports on other species [37,39,56], I proposed Hypothesis Ⅲ: in temperate-zone ESB amphibians, the threshold value for day 1 (i.e., T_1_ for males and t_1_ for females) is nearly equivalent to that for day 2 (i.e., T_2_ for males and t_2_ for females). Moreover, these values are nearly identical to the threshold value of the required PMAT (i.e., the threshold temperature inducing readiness for autumn migrations to the aquatic or underground wintering sites) before day 0 (i.e., T_0_ for males and t_0_ for females). Specifically, T_0_ ≈  T_1_ ≈  T_2_ and t_0_ ≈  t_1_ ≈  t_2_, as illustrated in Fig 5 and previously reported [51]. Furthermore, the results of this study demonstrates that all threshold values of *R. sakuraii* were higher in females than in males (Tables 1–6; T_0_ >  t_0_, T_1_ >  t_1_, and T_2_ >  t_2,_ in Fig 5). Therefore, *R. sakuraii* consistently exhibited “autumn protogyny and spring protandry” every year.

Based on Hypothesis Ⅲ, the results of this study, and the following evidence from previous studies on both (i) ESB migrations and (ii) autumn migrations, I propose Hypothesis Ⅳ: temperate-zone ESB amphibians show “autumn protogyny and spring protandry,” and that these contrasting behaviors are not independent but occur successively depending on the differences in the threshold temperature values (higher for females than for males) for hibernation (i.e., days F_0_ and F_1_ are earlier than M_0_ and M_1_, and day M_2_ is earlier than F_2_, depending on the threshold T >  t; Fig 5).

(i) Regarding ESB migrations, numerous studies have reported that the trigger for the start (i.e., the emergence from their hibernation) was the rising ambient daily water, soil, or air temperatures to specific threshold values [32–36]. In addition, many instances of protandrous behaviors have been previously reported in many species [3,4,19,37–44]. For example, Koskela and Pasanen [39], and Ryser [44] reported that male *Rana temporaria* emerged from hibernation and started ESB migrations on an average 0.8 and 2.0 days earlier than females, respectively. Slater et al. [43] and Gittins et al. [32] reported that male *Bufo bufo* started ESB migrations on an average of 2.0 days earlier than females, with threshold air temperatures (at dusk) of 3.8°C for males and 4.1°C for females. Thus, these reports suggest that emergence from hibernation is controlled by the ambient temperatures, and spring protandry is a common phenomenon exhibited by ESB amphibians and is induced by the difference of the threshold temperature values for emerging from hibernation.
(ii) Regarding autumn migrations to breeding sites, detailed studies are scarce. However, three studies focusing on the start and end conditions [37,39,51], and one report specifically addressing autumn protogyny exists [37]. Koskela and Pasanen [39] reported that autumn migrations of *R*. *temporaria* occurred on the first rainy days after the minimum air temperature had dropped to the first frost or near 0°C (i.e., PMAT), regardless of the air temperature on the start days. My previous study of *R. sakuraii* showed similar results, with the PMAT required to induce the start of autumn migration being approximately 5°C or lower [51]. Meanwhile, Hurlbert [37] was the only one to address the difference between the sexes. He reported that during the autumn migration, female newts started and arrived earlier than males. However, during the ESB migration, males started and arrived earlier than females. The onset of ESB migrations and the end of autumn migrations were strongly correlated with the ambient temperature. These findings closely parallel those of the present study, highlighting autumn protogyny and spring protandry, and their dependence on the ambient temperature for hibernation. Thus, it is inferred that the start of autumn migrations is also controlled by the ambient temperatures, with PMAT serving as the triggering factor (regardless of the air temperature on the start days). Importantly, the PMAT values are almost identical to the values (water or soil temperatures) that induce both the end of autumn migratory movements and the start of ESB migrations (i.e., both the entering and emerging for hibernation). Moreover, based on Hypothesis Ⅲ, if the factor inducing spring protandry is the difference between threshold temperature values for hibernation (i.e., T_2_ >  t_2_, in Fig 5), autumn migrations will inevitably be protandrous, contrary to spring protandrous migrations observed by both Hurbert [37] and the present study.

Meanwhile, among migratory birds (showing spring protandry), autumn protogyny has also been reported [59,60], and Mills [59] described the annual migratory behavior of birds as “spring protandry and autumn protogyny,” similar to the present study. Thus, migratory birds are also likely to show protogyny during autumn migrations (to wintering sites) and protandry during spring migrations (to breeding sites), which might depend on the difference in the threshold temperature for wintering (in effect, reproductive readiness).

### Adaptive significance of “autumn protogyny and spring protandry” for pairings of ESB amphibians

Notably, many amphibian studies do not account for the following three points regarding temperate-zone ESB amphibians: (1) significance of both autumn migrations and wintering conditions for their pairings, (2) the fact that many species perform “Early pairings”, and (3) the adaptive significance of autumn protogyny or protandry. These gaps likely stem from a scarcity of amphibian field researchers and the inherent challenges in studying amphibian autumn migrations, making such research relatively rare compared to studies on ESB migrations. Therefore, there is only one existing report on amphibian autumn migrations focusing on whether they exhibit protandry or protogyny [37]. Moreover, the adaptive significance, particularly of autumn protogyny or protandry, has not been extensively discussed prior to this study. However, a few reports on the adaptive significance limited to amphibian spring protandry are available [3,4].

**Fig 6:**
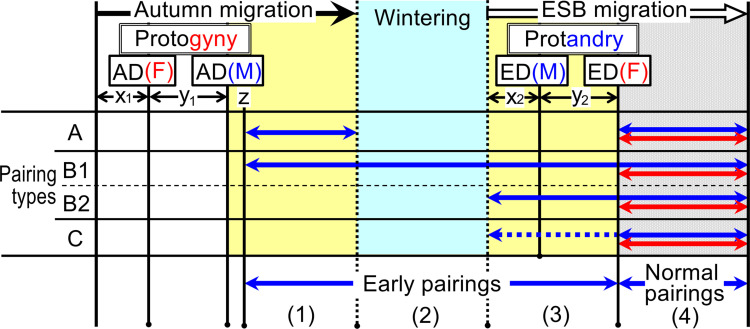
Pairing types (A, B, and C) by ESB amphibians, categorized by pairing time [“Early pairings” (1–3) and “Normal pairings” (4)] and fertilization modes (internal or external). Blue bold arrows: pairing time. Red bold arrows: spawning (egg-laying) time. x_1_: 0 or a few days. x_2_: 0 days. y_1_: and y_2_: a few days. z: the start day of male pairing behaviors triggered by male reproductive readiness. A. Internal fertilization species (most urodele species [24,25] and the tailed (copulating) frog [15,16,21]): These species are primarily aquatic hibernators, and pairings are performed in either autumn (1) or spring (4). Pairings are broken off immediately after the males deliver sperm to the females, with the females storing sperm until spawning in spring (4). B and C. External fertilization species: These pairings continue until females complete spawning. B (e.g., some *Bufo* spp. and some *Rana* spp [17–20,22,23,57].). B1) In these species, most pairings are “Early pairings”; however, some are “Normal pairings.” They are basically aquatic, non-torpid hibernators with group hibernation; B2) These species generally only perform “Normal pairings”; however, in the case of some local breeding sites, most or some pairings are performed as “Early pairings” due to group hibernation. C: Well-known type species that only perform “Normal pairings”. However, even among many C type species, some pairings would occur locally as “Early pairings” (shown the blue dotted line) due to group hibernation.

**(1) Significance of autumn migrations and wintering conditions for pairings of ESB amphibians.** Primarily, ESB amphibians should complete spawning immediately after emergence from hibernation, when it is still very cold, and their prey species have not yet emerged. Therefore, all the breeding individuals need to emerge at the breeding site at nearly simultaneously and complete breeding over a short period to subsequently experience a spring torpor period. To achieve this, they all migrate during autumn and hibernate at or close to their original aquatic breeding sites (i.e., Mode I, Fig 1). This strategy ensures they can accurately sense the suitable temperature of the breeding sites just before emergence from hibernation and to promptly arrive at the main breeding sites. If they fail to do so, individuals cannot emerge synchronously, and migrating long distances during their breeding period becomes too cold and risky. Therefore, both autumn migrations and “wintering conditions” are important for pairings of ESB amphibians, particularly for Mode I species, unlike Modes II and III species that migrate away from their breeding sites and hibernate at separate locations (Fig 1).
**(2) Types of “Early pairings”.** While many amphibian researchers typically assume that pairings in temperate-zone amphibians occur exclusively at the main breeding sites after emergence from hibernation (i.e., “Normal pairings” shown in Fig 6 (4)), ESB amphibians exhibit a notable occurrence of “Early pairings” (Fig 6 (1–3)). These “Early pairings” by ESB amphibians are classified into two types (A, B), based on pairing time and fertilization modes (internal or external), specifically whether pairings are maintained or not (Fig 6). Moreover, “Early pairings” are categorized into three stages: (1) during autumn or just before wintering, (2) during wintering (hibernation), and (3) immediately after emergence from hibernation but before emergence at the main aquatic breeding sites (indicated as stages 1–3 in Fig 6).

A. Internal fertilization species (Fig 6): These species are primarily aquatic hibernators and have the potential for pairing during both autumn and spring (as indicated in Fig 6 (1) and (4)), with females having the ability to store sperm. These pairings are broken off immediately after the males deliver sperm to the females, with only the females exhibiting spawning behavior during spring (as shown in Fig 6 (4)). The sperm delivered during the autumn pairing event is maintained within the females until spring. A1) Most urodele species, for instance the Salamandroidea comprises one of three suborders. These species account for approximately 90% of the order Urodela, and their representative families exhibit internal fertilization, classified as A type [24,25]. A2) Some anuran species, for instance the tailed frog *Ascaphus truei*, engages in copulation (pairing) during autumn (or spring), after which the females store sperm and spawn separately after hibernation [15,16,21]. B. Some anuran species also exhibit external fertilization through amplexus pairing (Fig 6). These pairings continue until the females complete spawning. In this B type, although most pairings are performed as ‘Early pairings,’ some pairings are consistently performed as ‘Normal pairings’ (Fig 6). B1). These species basically hibernate (aquatic and non-torpid) in groups under boulders in running streams. These pairings commence during autumn (as shown in Fig 6 (1)), and some or most females are amplexed by males, just before the beginning of hibernation and immediately after emergence from hibernation (e.g., *Bufo stejnegeri* [22]; *Bufo torrenticola* (unpublished data in Japan), and *Rana chensinensis* [20,23]), or during their autumn migration to their hibernation sites, as seen in *R. sakuraii* (57, refer to the first section of Results). B2). These species generally perform “Normal pairings” (Fig 6 (4)). However, in the case of some local breeding sites, most or some of its females are paired before emergence at the breeding sites (i.e., “Early pairings,” Fig 6 (3)), for example, *B. bufo* (84.4% [17,18]–94.3% [19] of females), *B*. *japonicus formosus*, and *R. ornativentris* (unpublished data in Japan). I hypothesize that these “Early pairings” of type B2 species are likely dependent on the local group hibernation, similar to B1 type species. Furthermore, even in well-known type species that only perform “Normal pairings” (i.e., C type in Fig 6), if the males hibernate close to females, the males have a high potential to successfully pair with females immediately after emergence from hibernation, similar to B2 species. Therefore, I hypothesize that, even among many C type species, some pairings would occur locally before emergence at the main breeding sites (indicated by the blue dotted line, Fig 6), albeit potentially going unnoticed.

Unlike types A and B, the Japanese giant salamanders engage in pairing and egg-laying during autumn, not early spring [26]. Additionally, unlike other migratory birds, waterfowl engage in pairing and copulation at wintering sites, and the sperm is maintained within the females until spring when the egg-laying occurs at the breeding sites following the spring breeding migrations [27–29]. Thus, for temperate-zone ESB amphibians (as well as waterfowl), both the autumn migrations and wintering conditions are more important for pairing compared to the ESB migrations and breeding-site conditions, respectively.

**Fig 7:**
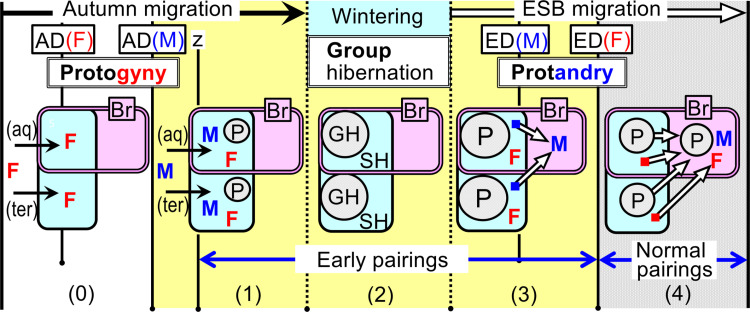
Adaptive significance of autumn protogyny (associated with group hibernation) and spring protandry for pairings of temperate-zone ESB amphibians. Pink rounded rectangles (double-lines with the initial Br): the aquatic breeding sites. Light blue rounded rectangles (solid-lines): the aquatic or terrestrial wintering (hibernation) sites. (aq): aquatic hibernators (Mode Ia, Fig 1); (ter): terrestrial hibernators (Mode Ib, Fig 1). z: the start day of male pairing behaviors triggered by male reproductive readiness. (i) In “Early pairings” (1–3): Although females are not in optimum reproductive conditions yet, if females arrive earlier (i.e., autumn protogyny) (0), males arriving later (completing reproductive conditions) can search more efficiently for females, and some males can pair before hibernation (1). Alternatively, even if the males cannot pair before hibernation, some or most males can hibernate close to females (i.e., group hibernation) (2). This increases the likelihood of males pairing with females immediately after emergence from hibernation, before emergence at the main breeding sites (3). (ii) In “Normal pairings” (4): The optimum time for female spawning is considerably shorter immediately after emergence from hibernation; therefore, if female arrival (emergence) is delayed (i.e., spring protandry), it would bring a group of males to the main breeding sites. This enables all females to promptly pair with the males at the optimum time. Furthermore, males remain at the breeding sites for longer durations, allowing them to fertilize multiple females and ensuring mating opportunities for all females, even without a male-biased sex ratio.

**(3) Adaptive significance of autumn protogyny and spring protandry for pairings of ESB amphibians.** I propose “the surefire pairing hypothesis” as the adaptive significance for the successively-arising contrasting behaviors of autumn protogyny and spring protandry in *R. sakuraii*, and other temperate-zone ESB amphibians. This hypothesis includes the three principal points: (i) Pairings of all females in the population are more reliably achieved in two stages: before and after emergence at main breeding sites, that is, “Early pairings” followed by “Normal pairings” (Fig 6). This approach ensures that even species primarily engaging in “Early pairings” (i.e., A and B types in Fig 6) also have some pairings occurring through “Normal pairings.” (ii) Pairing advantage are provided by autumn protogyny and its associated group hibernation during “Early pairings,” and by spring protandry during “Normal pairings.” This advantage benefits the sex that arrives later and searches for mates (males in autumn protogyny and females in spring protandry) (Fig 7). (iii) The sex with a higher threshold temperature for hibernation (females), which requires more time for reproductive readiness, gains an advantage in avoiding the risk of cold temperatures. This advantage is significant during both the end of autumn and the start of the ESB migration period.

The principal point (ii) of the “the surefire pairing hypothesis” is based on the following two characteristics: (1) Reproductive conditions (i.e., when is reproductive readiness completed? what is the duration of reproductive readiness? and which sex actively searches for mates?): The male reproductive readiness is usually in late autumn and continues through spring. Males can remain reproductively ready for many days and have the capacity to fertilize multiple females. In contrast, the female reproductive readiness requires much more time to achieve compared to males, and the optimum time for spawning is considerably shorter, lasting only a few days immediately after emergence from hibernation. Thus, from autumn to wintering periods, although activity levels in both sexes decrease due to lower ambient temperatures, some or most males can actively search for females and attempt to pair with them. Females, however, do not search for males until at least after emerging from hibernation. Conversely, during early spring, females must actively search for males within their brief optimal spawning window, while single males tend longer at the main breeding sites, waiting for females to arrive. (2) Wintering conditions (i.e., where and how do individuals hibernate? (e.g., group or solitary, aquatic or terrestrial, and torpid or non-torpid): Primarily, to breed immediately after emergence from hibernation, ESB amphibians tend to hibernate at or close to their aquatic breeding sites. This results in group hibernation, contrasting with Modes II and III species (Fig 1). Thus, many species of ESB amphibians are aquatic hibernators (e.g., most urodeles [37,52–55], *A. truei* [15,16], most brown *Rana* spp [20,23,36,39,46]., and some *Bufo* spp [22,61,62].). Moreover, several studies have reported that these species were non-torpid and reactive even during hibernation periods [20,23,36,39,57,62]. Subsequently, Stinner et al. [63] hypothesized that hibernating aquatic frogs are generally non-torpid (particularly when hibernating at running water sites that are non-torpid), and always reactive, based on research on aquatic hibernating frogs that remain reactive and move occasionally, even during cold periods. For instance, although *B. bufo* are generally terrestrial hibernators, in cold northern regions (Sweden), they were also found to hibernate in small streams, remaining non-torpid and reactive even at water temperatures of 1–4°C [62]. Thus, in ESB amphibians, this non-torpid aquatic group hibernation increases their potential to pair immediately after hibernation, before emerging at the main breeding sites, especially when hibernating in running water with non-torpid conditions.

Collectively, based on the aforementioned two characteristics—reproductive and wintering conditions—the principal point (ii) can be inferred as follows (Fig 7). First, to perform effectively “Early pairings,” males need to delay their arrival and hibernate close to the females, resulting in autumn protogyny and group hibernation (Fig 7 (0–4)). In autumn protogyny, females typically do not actively seek males due to their incomplete reproductive readiness. However, if females (the sought-after sex) arrive earlier (Fig 7 (0)), males (who arrive later and actively seek mates) can effectively search for females (Fig 7 (1)). Therefore, some males who have achieved complete reproductive conditions can pair with females before hibernation (Fig 7 (1)). Moreover, even if the males cannot pair with the females before hibernation, some or most males (in particular, aquatic hibernators) can hibernate close to females (i.e., group hibernation) (Fig 7 (2)). Furthermore, males that have participated in group hibernation have a higher potential to succeed in pairing with females immediately after emergence from hibernation, before emergence at the main breeding sites (Fig 7 (3)). In contrast, in the case of autumn protandry, males arriving earlier cannot efficiently search for females as they would have not yet arrived. Moreover, late-arriving females do not actively search for males. As a result, males have difficulty encountering females, and both sexes tend to hibernate solitarily. Secondly, to perform effectively “Normal pairings,” single males hibernating alone need to migrate earlier and wait for unpaired single females at the main breeding sites. Conversely, single females need to delay emergence, resulting in “spring protandry” (Fig 7 (4)). If the females were delayed in their arrival (emergence), it would bring a group of males to the main breeding sites, so that all the females arriving later can immediately encounter and pair with the males in the best timing for every female. Furthermore, males remain at the breeding sites for longer durations, allowing them to fertilize multiple females and ensuring mating opportunities for all females, even without a male-biased sex ratio. In contrast, in the case of spring protogyny, females arriving earlier do not encounter any males as they have not arrived yet. This delays the pairing process, potentially missing the optimal spawning timing for many females and reducing the success rate of spawning events.

Finally, regarding the adaptive significance of only the spring protandry in the temperate-zone ESB amphibians, I propose the “every female pairing hypothesis.” This hypothesis posits that spring protandry, where males arrive earlier, is crucial for ensuring that every female can pair with the males at their optimum spawning time, which is limited to only a few days immediately after emerging from hibernation, as detailed in the aforementioned descriptions. This explanation closely aligns with findings discussed by Semlitsch et al. [4].

In conclusion, temperate-zone ESB amphibians exhibit both “autumn protogyny and spring protandry,” ensuring secure pairings through two stages: before and after emergence at main breeding sites (i.e., “Early pairings” and “Normal pairings”). Autumn protogyny facilitates pairing in species that utilize internal fertilization (sperm stored in females) and anuran amplexus during late autumn or just before wintering>  group hibernation enables pairing immediately after emergence from hibernation>  spring protandry ensures pairing at the main breeding sites. This sequential advantage enhances breeding success by optimizing timing for both pairing and spawning at the population level.

## Supporting Information

S1 Table Investigated years during three life periods (autumn migration, hibernation, and ESB migration) and the corresponding number of captures. 1. The years and captured numbers presented in this table are the only data relevant to this paper. For instance, data regarding immature individuals and the spring torpor period are not included. 2. Determining the appropriate design, placement, and timing of net traps for both autumn and ESB migrations required several years of experimentation. This was necessary because the detailed migratory behaviors of this species were initially unknown. Various methods were tested, including trap design, distance between traps, and placement positions. As a result, systematic and optimized net-trap methods were established starting from the 2002–2003 season.
(PDF)

S2 Table Percentage of amplexed female *Rana sakuraii* captured by instream net-traps during the autumn instream movements in the breeding streams. These **v**alues represent the captured rate of amplexed females; however, it should be noted that during autumn, amplexed females typically do not migrate. Therefore, most pairs during the late autumn migration periods remain stationary and may not enter the instream net-traps.
(PDF)

S3 Table Percentage of amplexed female *Rana sakuraii* captured by hands or dip nets during the hibernation period. ^a^ The end day was defined as the day preceding the start day of the ESB migrations. These **v**alues do not represent the actual rate of amplexus because they were derived solely from sites where capture by hand was feasible. It is estimated that approximately 85% of adults hibernated in locations where researchers could not attempt capture, such as under large rocks or beneath boulders in deep stream pools, where individuals are presumed to remain stationary in groups or in amplectant pairs. Conversely, individuals captured by hand typically hibernate solitarily. Moreover, in late January, single males and females captured by hand or dip nets were observed to pair in amplexus when kept together in a bucket. All frogs were captured from different sites and were not recaptured during the same hibernation period, as surveys began at the most downstream stream section and gradually moved upstream. Approximately 120–150 m was surveyed per day.
(PDF)

S4 Table Percentage of amplexed, spent, and single gravid female *Rana sakuraii* captured by instream net-traps during the ESB migrations. ^a^ Regarding the study years (1993, 2006–2007, 2009–2012, and 2015–2017), instream net-traps were set from mid- or late January to the early morning of March 1 due to the unavailability of a special license from the Akigawa River Fishery Cooperative to set net-traps after March 1, which marks the opening day of the stream fishing season each year, despite ongoing migratory movements. In other study years, net-traps were set until the completion of migratory movements. ^b^ A notable characteristic of *R. sakuraii* is observed in the breeding period: while in most frog species the rate of single gravid (not spent) females decreases as it becomes late periods in the breeding season progresses, in *R. sakuraii*, this rate increases somewhat late in the breeding period (as shown at S4 Table: from 11.3% to 16.4%). This phenomenon arises due to the challenges that females of this species face during spawning. Normally, in most frog species, males release amplexus after successful female spawning. However, in *R. sakuraii*, if amplectant females cannot complete spawning over an extended period, amplectant males release them due to fatigue from prolonged amplexus, even though the females have not yet spawned. Therefore, toward the end of the breeding period of *R. sakuraii*, some single gravid females occur because males release them before successful spawning.
(PDF)
