# Supplementary material for: Autumn protogyny and spring protandry: Mechanisms and adaptive significance in a Japanese headwater frog, Rana sakuraii
Source: PLoS One. 2025 Apr 4;20(4):e0320076. doi: 10.1371/journal.pone.0320076 (PMC11970676; doi:10.1371/journal.pone.0320076)
Supplement: S1 Table — 1. The years and captured numbers presented in this table are the only data relevant to this paper. For instance, data regarding immature individuals and the spring torpor period are not included. 2. Determining the appropriate design, placement, and timing of net traps for both autumn and ESB migrations required several years of experimentation. This was necessary because the detailed migratory behaviors of this species were initially unknown. Various methods were tested, including trap design, distance between traps, and placement positions. As a result, systematic and optimized net-trap methods were established starting from the 2002–2003 season. (PDF) [file pone.0320076.s001.pdf]

S1 Table. Investigated years during three life periods (autumn migration, hibernation, and ESB migration) and the corresponding number of captures.

Year (Autumn to spring)	Autumn migration		Hibernation		ESB migration		Total
		No. captured		No. captured		No. captured	No. captured
1991–1992			1	1,156	1	3,103	4,259
1992–1993			2	1,747	2	9,510	11,257
1998–1999			3	2,774	3	5,158	7,932
1999–2000	1	1,666	4	3,817	4	3,117	8,600
2000–2001			5	1,686	5	10,415	12,101
2001–2002	2	1,673	6	745	6	7,075	9,493
2002–2003	3	5,363	7	2,666	7	7,731	15,760
2003–2004			8	1,629	8	4,200	5,829
2004–2005			9	1,124	9	2,846	3,970
2005–2006	4	3,807	10	2,454	10	7,354	13,615
2006–2007			11	845	11	3,655	4,500
2007–2008			12	1,178	12	2,634	3,812
2008–2009			13	1,105	13	6,499	7,604
2009–2010			14	1,179	14	4,219	5,398
2010–2011			15	1,191	15	3,420	4,611
2011–2012			16	1,357	16	3,304	4,661
2012–2013			17	560	17	1,037	1,597
2013–2014	5	702	18	938	18	1,180	2,820
2014–2015	6	792	19	548	19	1,231	2,571
2015–2016			20	255	20	1,388	1,643
2016–2017			21	443	21	741	1,184
Total	6	14,003	21	29,397	21	89,817	133,217
