# Supplementary material for: Autumn protogyny and spring protandry: Mechanisms and adaptive significance in a Japanese headwater frog, Rana sakuraii
Source: PLoS One. 2025 Apr 4;20(4):e0320076. doi: 10.1371/journal.pone.0320076 (PMC11970676; doi:10.1371/journal.pone.0320076)
Supplement: S2 Table — These values represent the captured rate of amplexed females; however, it should be noted that during autumn, amplexed females typically do not migrate. Therefore, most pairs during the late autumn migration periods remain stationary and may not enter the instream net-traps. (PDF) [file pone.0320076.s002.pdf]

S2 Table. Percentage of amplexed female *Rana sakuraii* captured by instream net-traps during the autumn instream movements in the breeding streams.

	No. of trapped females during the autumn migrations			Percentage of amplexed females	
	The whole females		Amplexed females		
	From the first arrival days	From the first amplexed days		From the first arrival days	From the first amplexed days
1999	586	396	26	4.4%	6.6%
2001	394	121	12	3.0%	9.9%
2002	1,605	827	36	2.2%	4.4%
2005	1,718	1,582	57	3.3%	3.6%
2013	172	170	20	11.6%	11.8%
2014	405	357	10	2.5%	2.8%
Total	4,880	3,453	161		
Mean				4.5%	6.5%
