# Supplementary material for: Autumn protogyny and spring protandry: Mechanisms and adaptive significance in a Japanese headwater frog, Rana sakuraii
Source: PLoS One. 2025 Apr 4;20(4):e0320076. doi: 10.1371/journal.pone.0320076 (PMC11970676; doi:10.1371/journal.pone.0320076)
Supplement: S3 Table — These values do not represent the actual rate of amplexus because they were derived solely from sites where capture by hand was feasible. It is estimated that approximately 85% of adults hibernated in locations where researchers could not attempt capture, such as under large rocks or beneath boulders in deep stream pools, where individuals are presumed to remain stationary in groups or in amplectant pairs. Conversely, individuals captured by hand typically hibernate solitarily. Moreover, in late January, single males and females captured by hand or dip nets were observed to pair in amplexus when kept together in a bucket. All frogs were captured from different sites and were not recaptured during the same hibernation period, as surveys began at the most downstream stream section and gradually moved upstream. Approximately 120–150 m was surveyed per day. (PDF) [file pone.0320076.s003.pdf]

S3 Table. Percentage of amplexed female *Rana sakuraii* captured by hands or dip nets during the hibernation period.

	Percentage of amplexed females to total females								
Year	December			January			February		
	1–10	11–20	21–31	1–10	11–20	21–31	1 Feb.–7 days before the end[a]	4–6 days before the end[a]	1–3 days before the end[a]
1991–1992					21.6		26.2	42.6	64.3
1992–1993			6.7	11.6	23.8	19.8			
1998–1999	7.1	11.9		24.0	20.7	28.6	25.0	51.3	57.1
1999–2000			7.9	13.3					45.5
2000–2001				9.5				33.3	
2001–2002			11.1	11.7	25.0	30.0			
2002–2003				13.4	18.8	20.0	21.9		
2003–2004						15.3	26.9	28.6	
2004–2005						15.6	23.5	25.5	
2005–2006		6.5	9.5	10.8		18.3	29.4		
2006–2007			20.0	12.1	14.6	21.7	25.0		
2007–2008				16.0	25.0	18.6	23.3		
2008–2009			23.1	22.2	19.3				
2009–2010			4.9		12.5		33.3		
2010–2011		4.5	9.1	18.8		16.7	18.2		
2011–2012				16.9	13.2	15.9	18.0		
2012–2013				10.7	13.0	22.2			
2013–2014	2.1	4.5							
2014–2015	2.7	6.6							
2015–2016		6.3							
2016–2017			12.5	13.2	12.3	16.1			
Mean	4.0	6.7	11.6	14.6	18.3	19.9	24.6	36.3	55.6
