# Supplementary material for: Autumn protogyny and spring protandry: Mechanisms and adaptive significance in a Japanese headwater frog, Rana sakuraii
Source: PLoS One. 2025 Apr 4;20(4):e0320076. doi: 10.1371/journal.pone.0320076 (PMC11970676; doi:10.1371/journal.pone.0320076)
Supplement: S4 Table — In other study years, net-traps were set until the completion of migratory movements. b A notable characteristic of R. sakuraii is observed in the breeding period: while in most frog species the rate of single gravid (not spent) females decreases as it becomes late periods in the breeding season progresses, in R. sakuraii, this rate increases somewhat late in the breeding period (as shown at S4 Table: from 11.3% to 16.4%). This phenomenon arises due to the challenges that females of this species face during spawning. Normally, in most frog species, males release amplexus after successful female spawning. However, in R. sakuraii, if amplectant females cannot complete spawning over an extended period, amplectant males release them due to fatigue from prolonged amplexus, even though the females have not yet spawned. Therefore, toward the end of the breeding period of R. sakuraii, some single gravid females occur because males release them before successful spawning. (PDF) [file pone.0320076.s004.pdf]

S4 Table. Percentage of amplexed, spent, and single gravid female *Rana sakuraii* captured by instream net-traps during the ESB migrations.

Year	Females (%) total (during setting net-traps)			Single gravid F / total F (%)		
	Amplexed	Spent	Single gravid	Start to peak	After peak	After 1 March[a]
1992	77.9	12.8	9.3	8.0	11.7	11.7
1993	78.3	12.9	8.8	8.3	9.9	
1999	77.0	5.1	17.9	16.8	19.2	16.6
2000	73.4	4.9	21.7	18.9	25.6	21.9
2001	73.3	15.0	11.7	9.8	14.3	10.3
2002	82.2	5.8	12.0	11.8	12.2	25.9
2003	80.7	8.9	10.4	9.7	11.2	15.4
2004	80.2	4.9	14.9	13.3	16.8	15.8
2005	85.2	5.6	9.2	8.1	11.0	19.3
2006	77.3	4.2	18.5	18.0	19.2	
2007	78.5	5.0	16.5	14.8	19.3	
2008	86.8	2.7	10.5	8.8	14.5	9.9
2009	78.6	2.0	19.4	18.0	21.9	
2010	85.9	0.7	13.4	12.6	16.4	
2011	87.9	2.9	9.2	8.1	11.6	
2012	91.3	4.2	4.5	2.4	7.5	
2013	87.8	1.3	10.9	10.9	11.0	18.1
2014	80.4	3.7	15.9	14.9	16.6	15.2
2015	80.7	8.9	10.4	7.2	13.4	
2016	74.5	11.8	13.7	8.5	8.7	
2017	92.9	0.0	7.1	7.9	3.4	
Mean	81.5	5.9	12.7	11.3[b]	14.1[b]	16.4[b]
[amplexed and spent] F			87.3	88.7	85.9	83.6
